# Blockchain-Based Secure Storage and Access Control Scheme for Supply Chain Ecological Business Data: A Case Study of the Automotive Industry

**DOI:** 10.3390/s23167036

**Published:** 2023-08-09

**Authors:** Songjiang Li, Tao Zhou, Huamin Yang, Peng Wang

**Affiliations:** 1 College of Computer Science and Technology, Changchun University of Science and Technology, Changchun 130022, China; lsj@cust.edu.cn (S.L.); yhm@cust.edu.cn (H.Y.); wangpeng@cust.edu.cn (P.W.); 2 Chongqing Research Institute, Changchun University of Science and Technology, Chongqing 401120, China

**Keywords:** attribute-based access control, blockchain, automotive supply chain, secure storage, Hyperledger Fabric, smart contract, auditability

## Abstract

The reliable circulation of automotive supply chain data is crucial for automotive manufacturers and related enterprises as it promotes efficient supply chain operations and enhances their competitiveness and sustainability. However, with the increasing prominence of privacy protection and information security issues, traditional data sharing solutions are no longer able to meet the requirements for highly reliable secure storage and flexible access control. In response to this demand, we propose a secure data storage and access control scheme for the supply chain ecosystem based on the enterprise-level blockchain platform Hyperledger Fabric. The design incorporates a dual-layer attribute-based auditable access control model for access control, with four smart contracts aimed at coordinating and implementing access policies. The experimental results demonstrate that the proposed approach exhibits significant advantages under large-scale data and multi-attribute conditions. It enables fine-grained, dynamic access control under ciphertext and maintains high throughput and security in simulated real-world operational scenarios.

## 1. Introduction

With the rapid progress of globalization and digitization, traditional supply chain systems are encountering escalating challenges. Taking the automotive industry as a case in point, numerous large-scale automobile manufacturers rely on multiple suppliers worldwide to mitigate manufacturing costs [1]. This not only amplifies the geographic dispersal of the supply chain but also introduces intricate interrelationships. In comparison to other sectors, the automotive industry’s supply chain is characterized by slow response, deficient integration, and limited visibility [2]. The root cause underlying this predicament lies in the opaqueness of transactions among entities within the supply chain, leading to a dearth of trust and coordination among its participants [3].

Fortunately, with the advent of the Industry 4.0 era, emerging technologies such as blockchain, the Internet of Things (IoT) [4,5], and artificial intelligence (AI) are transforming traditional supply chain operations. These technologies have the potential to address the aforementioned challenges faced by traditional supply chains and have a positive impact on sustainability. Blockchain, as a disruptive distributed ledger technology [6,7], offers opportunities for end-to-end supply chain visibility and traceability due to its consensus mechanism, decentralization, system and data reliability, and information transparency [7,8,9]. In addition, the immutability recorded in the blockchain ledger ensures the authenticity of historical operational information within the supply chain. In today’s complex supply chain networks, blockchain in the supply chain ensures the best solution for transmitting transactions, promoting traceability, transparency, security, and resource management within the network [10].

Many scholars have already discussed the application of blockchain in automotive supply chain information sharing and traceability [11,12,13] and demonstrated the effectiveness of such solutions. However, as privacy and information security issues become more prominent, especially after the enactment of regulations such as the General Data Protection Regulation (GDPR) in the European Union (EU) [14,15], the challenge of achieving blockchain-based supply chain information sharing while protecting sensitive corporate information (e.g., transaction records, product batch information, logistics information) becomes crucial [16]. Moreover, existing solutions mostly directly store data on the blockchain, which undoubtedly brings tremendous storage pressure and reduces overall performance in large-scale data sharing scenarios. It is no longer able to meet the requirements for highly reliable secure storage and fine-grained access control mechanisms. Therefore, this study aims to contribute to the literature on blockchain in the automotive supply chain through addressing the following research questions:

RQ1. How to address the storage pressure of blockchain under large-scale data volume and establish a secure and efficient decentralized automotive supply chain data storage and sharing solution with privacy protection mechanisms?

RQ2. How to achieve one-to-many fine-grained access control to ensure authorized participants’ compliant access to specified business data?

Through addressing these research questions and considering the characteristics of the automotive supply chain, we propose a blockchain-based secure storage and access control scheme for business data in the supply chain ecosystem. The integration of a blockchain-based secure storage and access control scheme can provide data privacy protection, alleviate the storage pressure of blockchain, and offer fine-grained access control mechanisms. This contributes to enhancing the sustainability, reliability, and security of the supply chain ecosystem.

From the abovementioned discussions, the main contributions of this article are as follows:

1. To present a proposed scheme for storing and sharing business data in the context of data-sharing within the automotive supply chain. The scheme involves encrypting the data using a hybrid encryption algorithm and storing it in IPFS. On the blockchain, the scheme enables the authorization and sharing of key ciphertext and data resource addresses. Data are encrypted and stored in a decentralized manner, providing a high degree of security.
2. To propose a dual-layer attribute-based auditable access control model. The first tier utilizes the CP-ABE algorithm for data access control, while the second tier employs smart contract optimization and coordination for access control, ensuring the auditability of access records. This method enables flexible access control policies under ciphertext, reduces the risk of plaintext transmission, and guarantees the privacy of data sharing processes.
3. To put forward four smart contracts based on the Hyperledger Fabric platform, accompanied by their performance evaluation. The first smart contract manages data resources, the second one handles access control policies, the third is responsible for access control of data requests, and the fourth one enables access record auditing. These smart contracts exhibit replicability and portability in other data-sharing scenarios within the manufacturing industry.

The subsequent sections of this paper are structured as follows. Section 2 introduces the related work. Section 3 provides an overview of data-sharing scenarios for the automotive supply chain business, and Section 4 discusses the scheme for storing and sharing business data, the dual-layer auditable access control model, and the implementation of smart contracts. Section 5 presents the experimental evaluation and analysis of the results. Finally, in Section 6, we conclude and discuss the paper.

## 2. Related Work

In this section, we have reviewed the latest research in the relevant field, with the main aim of addressing the challenges of data storage and access control in blockchain-based automotive supply chains. We begin with a comprehensive review of blockchain applications in automotive supply chain systems, followed by a thorough discussion of access control schemes based on blockchain technology.

### 2.1. Blockchain-Based Supply Chain Scheme for Automotive

Patro et al. [17] proposed a blockchain-based scheme to address the deficiencies in transparency, traceability, auditing, and trust in the automotive supply chain’s product recall process. Their approach combines the Ethereum blockchain with decentralized storage IPFS to handle large-scale data storage challenges, enabling automobile manufacturers to achieve end-to-end information visibility during product recalls. Chen et al. [18] introduced a blockchain and smart contract-based framework for the automotive supply chain and specifically designed the communication process and algorithms within the blockchain. Their framework aims to mitigate security risks stemming from vehicle and component defects, as well as automotive fraud issues such as information asymmetry between suppliers and consumers. However, this scheme overlooks the challenges of blockchain storage bottlenecks and access control in one-to-many data-sharing scenarios. Zafar et al. [19] presented a blockchain-based automotive supply chain framework using Hyperledger Fabric, with a focus on achieving secure and efficient supply chain management in the automotive industry. However, detailed explanations regarding data access control were lacking. Ashraf et al. [20] proposed a software architecture that combines blockchain with Internet of Things (IoT) devices, enabling the traceability of general products throughout the supply chain, involving multiple levels of suppliers, logistics, manufacturers, and end customers. Their approach provides a digital solution for a generic supply chain that improves source traceability and operational reliability, highlighting the application potential of combining blockchain and supply chain. Guo et al. [21] proposed an information management framework BC4Regu based on BCT and IoT, aiming at improving the supervision of information transparency in SCF business processes. Their scheme provides a new idea for solving the problem of information asymmetry in supply chain financial transactions. Chou et al. [22] ensured confidential information sharing between enterprises, partners, and competitors in the supply chain while enhancing supply chain transparency through the construction of a multi-asset, multi-chain framework and the utilization of Hyperledger Fabric to establish a permissioned blockchain network. Their research provides a new solution for applying blockchain technology to improve information sharing in dynamic supply chains, especially with partners and competitors, but lacks considerations for data security.

### 2.2. Access Control on the Blockchain Scheme

Han et al. [23] proposed an auditable access control model based on blockchain that manages access control policy networks for private data based on attribute-based access control (ABAC) models. Their approach utilizes request records, response records, and access records stored in the blockchain. However, encryption protection for private data was not addressed. Shammar et al. [24] introduced an attribute-based access control model using blockchain (ABAC-HLFBC), which grants access permissions based on target-provided attributes, eliminating the need for access control lists (ACLs) or assigning roles to all system users. Liu et al. [25] proposed a fine-grained access control (FGAC) framework based on blockchain Hyperledger Fabric for supply chain data-sharing. The framework enhances role-based access control (RBAC) through assigning different attribute keywords to different user types. Adopting the ABAC model allows for more flexible and fine-grained dynamic management of privileges. The model is more suitable for practical application than the current mainstream RBAC model. Gao et al. [26] integrated role-based access control policies (RBACPs) into the design and implementation of a Fabric blockchain-based port supply chain system (Fabric-PSChain), bolstered by the inclusion of regulatory nodes to enhance data trustworthiness and security. Zhao et al. [27] proposed a decentralized attribute-based fine-grained access control scheme. In this scheme, encrypted data are stored on IPFS, the hash value is stored on Hyperledger Fabric for data-sharing, and a linear integer secret-sharing algorithm is employed to achieve symmetric key sharing among multiple attributes, ensuring key security. Lastly, Li et al. [28] combined the traditional RBAC model with the attribute-based access control (ABAC) model for managing access control in the medical device supply chain. Their objective was to provide fine-grained and dynamic permission management. However, it lacks the protection of data privacy.

Based on the aforementioned related work, current blockchain research in the automotive supply chain domain mostly either neglects discussing access control and the bottleneck of large-scale data storage when maintaining the blockchain ledger or adopts access control models that do not consider privacy protection. This could potentially lead to the leakage of sensitive data from all parties during the sharing process, thereby reducing the overall information security of the blockchain system. There is an urgent need for a comprehensive solution that can provide data privacy and security protection in large-scale data scenarios while possessing fine-grained access control mechanisms.

## 3. Data-Sharing Scenarios

The automotive supply chain is a multifaceted system involving numerous stages and stakeholders. Within this context, the reliable storage and sharing of business data hold significant importance [29]. Ranging from raw material procurement to component production, quality inspection, assembly, product sales, and after-sales maintenance, the business data generated at each stage are invaluable resources for the relevant participants. Furthermore, they are vital for facilitating the efficient operation of the supply chain. Secure information sharing between different stages is indispensable for promptly adjusting production plans, addressing production anomalies, enhancing product quality, and effectively responding to changes in market demand [30].

To illustrate, when a component manufacturer produces defective parts, it is imperative for the original equipment manufacturer (OEM) to expeditiously receive this information and take appropriate measures to prevent the propagation of product quality issues throughout the supply chain. Nevertheless, as the volume of data increases and the complexity of information sharing grows, supply chain managers are increasingly concerned with data management and privacy protection. During the data-sharing process, safeguarding proprietary business information is crucial. This necessitates the formulation of suitable data access policies and privacy protection measures based on the flow of business data to mitigate the risks of data leakage and misuse. Through conducting an analysis of the information flow in the automotive supply chain process, as depicted in Table 1, we selectively extract representative business data from different stages of the supply chain’s lifecycle and determine the subsequent data flow. Business information analysis serves as the foundation for subsequent data storage and sharing schemes, thereby ensuring the secure circulation of data throughout the data-sharing process.

## 4. Scheme Model Design

In this section, we propose a business data secure storage and access control scheme in a data-sharing scenario within the automotive manufacturing supply chain. The design and architectural details of this scheme will be fully elaborated in the following content.

Table 2 illustrates the key symbols used in our scheme and their respective meanings.

### 4.1. Business Data Storage Sharing Scheme

In our business data storage and sharing scheme, there are five main entities: Users, Certificate Authorities, Key Management Authorities, Fabric Blockchain, and IPFS.

- **Users:** Users represent the personnel responsible for managing the business data of various enterprises within the supply chain. They can fulfill both the roles of data owners (DOs) and data requesters (DRs). DOs encrypt the original data and securely store it in IPFS. The data resource address and decryption key are then encrypted using an attribute encryption algorithm and uploaded to the blockchain. Access control policies are optimized and coordinated through smart contracts. DR represents users who request data resources. Once their identity attributes satisfy the access control policies set by the DO, they can obtain the ciphertext of the data resource address and decryption key. Through utilizing the system’s public key and their own attribute private key, they can recover the data resource address and symmetric key, enabling them to retrieve the original business data from IPFS through decryption. Furthermore, the DO has the ability to establish access control policies for private data and perform auditing of access records through smart contracts.
- **Certificate Authority (CA):** The primary role of the CA is to issue digital certificates to participants within the network. These digital certificates serve to verify the identity of participants and facilitate encrypted communication. During the creation of digital certificates, the CA verifies the identity of the applicant and embeds their identity and attribute information into the digital certificate, granting them the corresponding identity credentials.
- **Key Management Authority (KMC):** The KMC is responsible for securely managing the system’s master key and generating user private keys. Additionally, it provides CP-ABE encryption and decryption services for user data.
- **Fabric Blockchain:** Fabric Blockchain is accountable for storing the encrypted data resource addresses and keys under the DO chain. It optimizes and coordinates access control policies through smart contracts and offers auditable data request records for the DO.
- **InterPlanetary File System (IPFS):** IPFS provides a decentralized data storage platform for the DO, ensuring a distributed and secure storage scheme.

The proposed business data storage and sharing scheme employs a hybrid encryption mechanism to guarantee data security and encryption efficiency. Initially, users encrypt their business data using the AES symmetric encryption algorithm, which boasts rapid encryption/decryption speeds and a high level of security. This encrypted data are then stored in IPFS. This approach effectively mitigates the storage burden on the blockchain and safeguards against data loss from a single node. Subsequently, the AES key is encrypted using the CP-ABE algorithm and uploaded to the blockchain for one-to-many authorization sharing. Through integrating a dual-layer auditable access control model, the scheme achieves decentralized privacy storage and ensures the immutability of business data, promoting trustworthy sharing and collaboration among enterprises.

**Figure 1:**
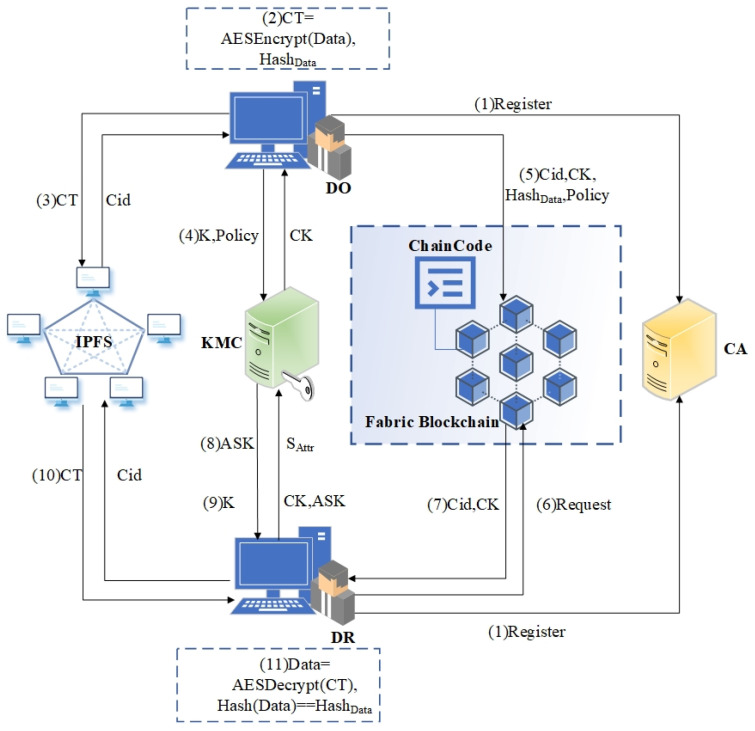
Architecture diagram of storage sharing scheme.

As shown in Figure 1, the workflow is as follows:

1. The DO and DR register their identities with the CA and submit their identity and attribute information to obtain digital certificates.
2. The DO calculates the hash value of the business data using the SHA-256 algorithm, referred to as Hash_Data_, and then encrypts the business data using the AES symmetric encryption algorithm, referred to as CT.
3. The DO uploads the CT to IPFS and obtains its content identifier, Cid.
4. The DO formulates the attribute encryption control policy, EncPolicy, and submits it along with the AES key to the KMC, resulting in the ciphertext CK embedding the access policy for the AES key.
5. The DO uploads the data’s access control policy, Cid, CK, and Hash_Data_ to the Fabric Blockchain through invoking the policy management contract and the resource management contract.
6. The DR initiates an access request to the business data owned by the DO through invoking the data access contract. The access management contract determines whether the DR’s attributes satisfy the access control policy for the requested data and records the access operation on the Fabric Blockchain.
7. If the DR’s attributes satisfy the policy, the Fabric Blockchain returns the Cid and CK of the requested data.
8. The DR uploads their attribute set S_Attr_ to the KMC and obtains the user attribute private key, ASK.
9. The DR uploads the CK and ASK to the KMC to perform the decryption operation and obtains the AES key, K.
10. The DR searches for the CT in IPFS based on the content identifier, Cid.
11. The DR decrypts the CT using the AES key, K, to obtain the original business data and verifies the integrity and non-tampering of the data through comparing Hash (Data) with Hash_Data_ obtained from the Fabric Blockchain.

### 4.2. Dual-Layer Auditable Access Control Model

**Figure 2:**
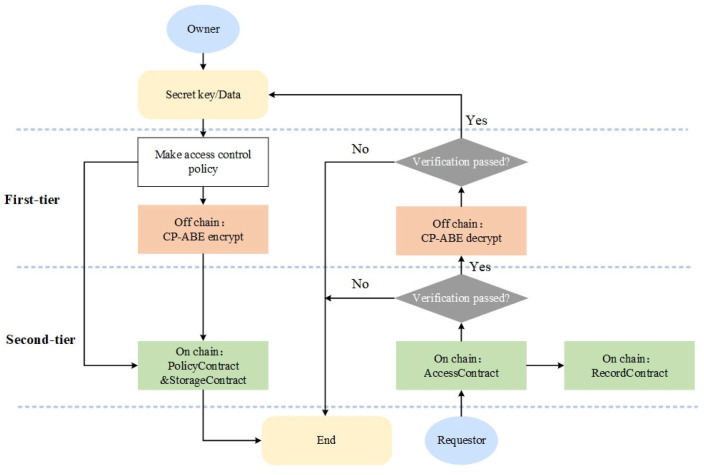
Dual-layer auditable access control model.

Due to the inclusion of corporate trade secrets and sensitive information, as well as explicit data flow, corporate users urgently need to establish flexible access control policies for shared data. In view of this, we propose an attribute-based dual-layer auditable access control model. The access policy determined by the data flow consists of two parts: an off-chain entity attribute policy **<EncPolicy>** and an on-chain global attribute policy **<Policy>**, as shown in Figure 2. Once the access policy is formulated, the DO coordinates access control through the off-chain CP-ABE attribute encryption algorithm and the on-chain auditable access contract. This approach ensures auditable access control under ciphertext, thereby mitigating the risks associated with cleartext transmission. In addition, it has a more flexible and safer access control mechanism than using the CP-ABE algorithm completely.

The supply chain scenario depicted in this model involves OEM A’s intention to share vehicle production business information with logistics provider B and distributors C and D, facilitating subsequent vehicle transportation and sales. To safeguard the legitimate dissemination of such business information, OEM A initiates the process through encrypting the data and securely storing it in IPFS. They proceed through formulating an access policy that aligns with the data flow and accomplishing the encryption of the AES key, subsequently uploading it to the blockchain. In due course, logistics provider B and distributors C and D, having met the necessary policy prerequisites, can retrieve the ciphertext of the key via the blockchain and decrypt it utilizing their respective attribute private keys. This enables them to access the AES key and decrypt the pertinent business data.

#### 4.2.1. Off-Chain Attribute-Based Encryption

**Figure 3:**
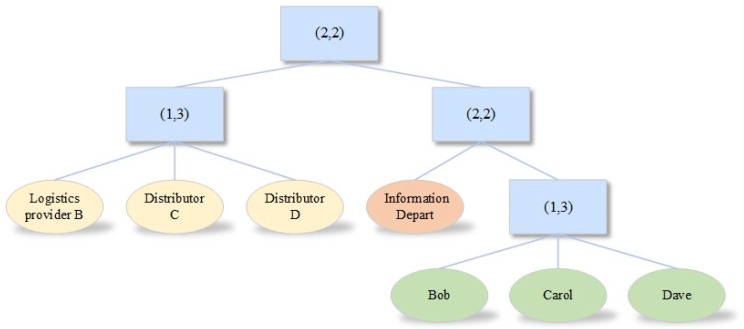
Access control policy tree.

The first layer of the model utilizes an attribute-based encryption algorithm to secure the AES key. The attribute encryption algorithm used in this model is based on the scheme construction of Bethencourt et al. [31]. The access control policy is represented through an access tree structure. For the sake of illustration, let us consider Alice, a staff member in the production department at OEM A, who is responsible for uploading production business data. Bob, a member of the information department at Logistics Company B; Carol, a member of the information department at Distributor C; Dave, a member of the information department at Distributor D; and Eve, a member of the information department at Distributor E, are requesting the data. Alice formulates the access control structure, known as EncPolicy, based on three attribute categories: company, department, and role, as depicted in Figure 3.

In Figure 3, attribute values are represented by leaf nodes, while non-leaf nodes are denoted by (t,n) thresholds. These thresholds indicate that the node has n child nodes, and among them, t child nodes must satisfy the subtree policy. Through recursively traversing from bottom to top, if the attribute set of the decryption party fulfills all the subtree policies, the ciphertext can be decrypted. It is evident that the attribute sets possessed by Bob, Carol, and Dave, among the data requesters, all comply with Alice’s access control policy and can be decrypted using their respective private keys. However, Eve’s attribute set fails to meet the policy requirements, rendering her unable to decrypt the data. Furthermore, companies can establish more intricate access control policies tailored to their specific business needs.

The CP-ABE utilizes the following four algorithm processes:

1. Setup→(APK, AMK): The algorithm executes during the initialization phase of the key management authority, generating the system public key (APK) and the system private key (AMK). To accomplish this, the algorithm chooses a bilinear group $G_{0}$ of prime order p, with g as the group’s generator. The size of the group is determined by the system security parameter λ. $\alpha ,\beta \in Z_{p}$ are the two encrypted exponents randomly selected by the algorithm. The resulting APK and AMK are outlined below:
  (1) $$APK=\left(G_{0},g,h=g^{\beta },f=g^{1/\beta },e{\left(g,g\right)}^{\alpha }\right)$$
  (2) $$AMK=\left(\beta ,g^{\alpha }\right)$$
2.  Encrypt(APK, K, EncPolicy)→(CK): The DO utilizes this algorithm to encrypt the AES key and incorporate the access policy <EncPolicy> into the ciphertext CK. Initially, the algorithm assigns a polynomial $q_{x}$ to each node in <EncPolicy>, following a top-down approach, where the degree of $q_{x}$ is defined as $d_{x}=t_{x}-1$. Commencing from the root node R, a random selection $s\in Z_{p}$ is made; let $q_{R}\left(0\right)=s$. For the remaining nodes x, let $q_{x}\left(0\right)=q_{parent\left(x\right)}(index\left(x\right))$. The leaf node set is denoted as Y. The ciphertext, which encapsulates the access policy <EncPolicy>, is represented as follows:
  (3) $$\begin{array}{l} CK=(EncPolicy,\tilde{C}=Ke{\left(g,g\right)}^{\alpha s},\\ \;\;\;\;C=h^{s},\forall y\in Y:C_{y}=g^{q_{y}(0)},C_{y}^{\prime }=H{\left(\mathrm{att}(y)\right)}^{q_{y}(0)}) \end{array}$$
3. $\;KeyGen\left(AMK,\;S_{Attr}\right)\to \left(ASK\right)$: This algorithm is employed to generate a private key for data requesters. It takes the user’s attribute set S_Attr_ as the input and produces the user’s private key ASK embedded with the corresponding attribute set. In this context, $r\in Z_{p}$ denotes a randomly selected number by the algorithm. For each attribute $j\in S_{Attr}$, a random selection $r_{j}\in Z_{p}$ is performed. Consequently, the user’s private key is determined as follows:
  (4) $$ASK=\left(D=g^{(\alpha +r)/\beta },\forall j\in S_{\mathrm{Attr}}:D_{j}=g^{r}\cdot H{\left(j\right)}^{r_{j}},D_{j}^{\prime }=g^{r_{j}}\right)$$
4. Decrypt(CK, ASK)→(K): The DR utilizes this algorithm to decrypt the ciphertext. The algorithm employs the user’s ASK to decrypt the CK and obtain the plaintext AES key, K. Successful decryption is contingent upon the user’s attribute set S_Attr_ satisfying the access structure. Primarily, let us define the recursive algorithm DecryptNode(CK, ASK, x):
  When x is a leaf node, let i=att(x); if $i\in S_{Attr}$_,_ then:
  (5) $$\begin{array}{cc} DecryptNode\left(CK,ASK,x\right)= & \frac{e\left(D_{i},C_{x}\right)}{e\left(D_{i}^{\prime },C_{x}^{\prime }\right)}\\ = & \frac{e\left(g^{r}\cdot H{\left(i\right)}^{r_{i}},h^{q_{x}\left(0\right)}\right)}{e\left(g^{r_{i}},H{\left(i\right)}^{q_{x}\left(0\right)}\right)}\\ = & e{\left(g,g\right)}^{rq_{x}\left(0\right)} \end{array}$$
  When x is not a leaf node, calculate $F_{z}=DecryptNode(CK,\;ASK,\;z)$ for all child nodes z of x. Let $S_{x}$ be the set of non-leaf node child nodes z of the size of any node threshold $t_{x}$. If $F_{z}$ has a value in the set, then the set exists. Calculate the following:
  (6) $$\begin{array}{cc} F_{x}= & \prod_{z\in S_{x}}F_{z}^{\Delta_{i,S_{x}^{\prime }}\left(0\right)},where\;i=index\left(z\right),S_{x}^{\prime }=\left\{index\left(z\right):z\in S_{x}\right\}\\ = & \prod_{z\in S_{x}}{\left(e{\left(g,g\right)}^{r\cdot q_{z}\left(0\right)}\right)}^{\Delta_{i,S_{x}^{\prime }}\left(0\right)}\\ = & \prod_{z\in S_{x}}{\left(e{\left(g,g\right)}^{r\cdot q_{parent\left(z\right)}\left(\mathrm{index}\left(z\right)\right)}\right)}^{\Delta_{i,S_{x}^{\prime }}\left(0\right)}\\ = & \prod_{z\in S_{x}}e{\left(g,g\right)}^{r\cdot q_{x}\left(i\right)\cdot \Delta_{i,S_{x}^{\prime }}\left(0\right)}\\ = & e{\left(g,g\right)}^{r\cdot q_{x}\left(0\right)} \end{array}$$
  If S_Attr_ satisfies the access structure <EncPolicy>, let
  (7) $$\begin{array}{cc} A= & DecryptNode\left(CK,ASK,r\right)\\ = & e{\left(g,g\right)}^{rs} \end{array}$$
  Finally, the plaintext K is calculated as follows:
  (8) $$\begin{array}{cc} \tilde{C}/\left(e\left(C,D\right)/A\right)= & \tilde{C}/\left(e\left(h^{s},g^{\left(\alpha +r\right)/\beta }\right)/e{\left(g,g\right)}^{rs}\right)\\ = & K \end{array}$$

#### 4.2.2. On-Chain Auditable Access Control

**Figure 4:**
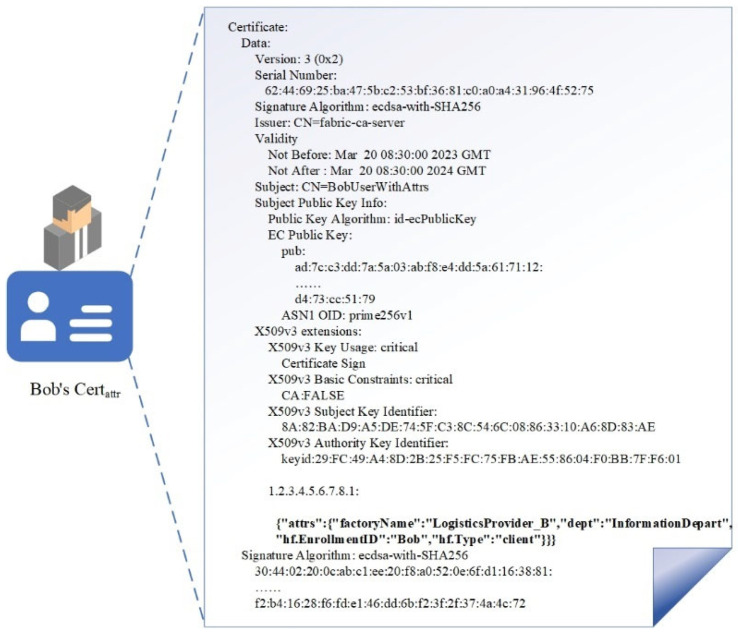
X.590 digital certificate with attribute information.

The second layer of the model consists of three parts: **{Storage}**, **{Policy}**, and **{Record}**. After the DO uploads the data (referring to the off-chain address and the ciphertext of the decryption key) to the blockchain, they can create attribute-based access control policies for the data. The on-chain smart contract automatically enforces access control based on the policies and the digital certificates of the DR which include identity attributes. This ensures the security and controllability of data-sharing on the blockchain. The X.509 certificate containing attributes is shown in Figure 4. When data are requested, the request record will be written into the ledger, creating an immutable access record that provides auditability of data access actions, meeting the requirements of data regulation. For users who violate the request for data, the data owner can revoke their access permissions through modifying the access policies to protect the privacy and security of enterprise data. The specific definitions of each part on the chain are as follows:

**{Storage} = {DataId, Owner, DataType, Cid, Ck, Hash_Data_}**. Storage represents the definition of data resources in the ledger. DataId is the unique identifier of the data, Owner is the identifier of the data owner, DataType is the data type, Cid is the content address of the data in IPFS, CK is the ciphertext of the symmetric key encrypted with off-chain properties, and Hash_Data_ is the hash value of the original data.

**{Policy} = {PolicyId, Status, Owner, Attributes}**. Policy represents the definition of access policies in the ledger and has a one-to-one relationship with {Storage}. PolicyId is the unique identifier of the policy, and Status indicates the policy status, with a value of 0 or 1 representing whether the policy is enabled. Owner is the identifier of the data owner. Attributes = {SA, DA, TA} are attribute definitions. The contract relies on Attributes to generate access control rules, and the categories of attributes included are defined as follows.

SAs (Subject Attributes): Used to identify and authenticate the visitor’s identity. The subject attributes include the information shown in Table 3.

DAs (Data Attributes): Used to identify the attributes of data resources. The data attributes include the information shown in Table 4.

TAs (Time Attributes): Used to define rules for accessing on-chain data within a specific time range. The time attributes include the information shown in Table 5.

Attributes are defined in JSON format, as shown in Table 6. DO can upload and manage policies through the PolicyContract. When a DR accesses the data, they can initiate an access request to the blockchain through the client. The AccessContract will determine the legitimacy of the request based on the access policy of the requested data and perform subsequent actions based on the determination result.

**{Record} = {RecordId, RequestLog}**. Record represents the definition of access records in the ledger and has a one-to-one relationship with {Storage}. RecordId is the unique identifier of the data record, and RequestLog represents the operational records of a DR on {Storage}. When a DR initiates a request, the RecordContract automatically appends the access record to the specified data’s RequestLog, ensuring transparent and auditable access records. The information included in the RequestLog is shown in Table 7.

### 4.3. Smart Contract Construction

In this section, we propose four smart contracts, namely StorageContract, PolicyContract, AccessContract, and RecordContract, to facilitate the specific implementation of auditable access control on the blockchain. Through the collaborative efforts of these smart contracts, on-chain data-sharing and auditable fine-grained access control are realized. DOs have the ability to define access policies, while DRs can submit access requests. The access contract plays a crucial role in validating and controlling data access permissions. Additionally, the record contract meticulously tracks all access events and logs, providing robust evidence for the purposes of auditing and tracing data-sharing activities. This decentralized mechanism for data-sharing and access control ensures enhanced data privacy and security for users. Subsequent sections will elaborate on smart contracts and their primary functionalities.

The **StorageContract** is responsible for managing the storage and updating of data resources on the blockchain. It encompasses the following primary functionalities: The ‘addData()’ function enables the DO to add data resources to the blockchain, facilitating sharing with other users. Before execution, the function performs a check to avoid duplicate entries. The ‘updateData()’ function enables the DO to modify existing data resources to maintain their currency. Before performing the operation, it is necessary to verify that the user’s client is the rightful owner of the data. The ‘getOwnData()’ function enables the DO to access the data they have stored for further processing or sharing.

The **PolicyContract** is responsible for managing data-sharing policies on the blockchain, encompassing the following key functionalities: The ‘addPolicy()’ function allows the DO to add a data-sharing policy through uploading pre-defined data access policies to the blockchain. The ‘getPolicy()’ function is called internally via the accessControl() function of the AccessContract to retrieve the access policy for the specified data, facilitating the determination of access control. The ‘queryPolicy()’ function allows the DO to query the defined data-sharing policies, providing them with an understanding of the access control rules for specific data. The ‘updatePolicy()’ function allows the DO to dynamically update the defined data-sharing policies, enabling it to adjust data access permissions in real-time. In the case of malicious requests, the DO has the ability to revoke access to the data. It is important to emphasize that this function necessitates verification of the user’s client as the data owner before execution to prevent malicious users from changing data access policies and compromising the security of data-sharing. The ‘changeStatus()’ function allows users to change the status of a policy, including enabling or disabling a data-sharing policy.

The **AccessContract** is responsible for managing data access requests and implementing access control. It encompasses the following primary functionalities: The ‘requestData()’ function is used by the DR to submit data access requests and retrieve the desired data. Before granting access, the function calls accessControl() to check that the user has the required permissions. It also calls the writeAccessRecord() function within the RecordContract contract to record the access outcome in the ledger, creating an immutable access record to facilitate subsequent data auditing and the adjustment of access control policies. The ‘accessControl()’ function is used to control access to data requests. It verifies the legitimacy of the data request and makes an authorization decision based on the access policy associated with the requested data and the set of identity attributes contained in the requester’s client digital certificate. The function returns the determination result based on the policy, as shown in the pseudo-code provided in Algorithm 1.

The **RecordContract** is tasked with documenting access control-related events and logs. It encompasses the following key functionalities: The ‘writeAccessRecord()’ function captures access records. This function is used to meticulously record data access events, including comprehensive details of the access request and its outcome. The ‘getAccessRecord()’ function is used by the DO to access data access records. This function allows the DO to retrieve access events and logs associated with shared data, facilitating auditing and changes to permissions.
**Algorithm 1:** AccessContract.accessControl()Require: ac *MyContract, dataId, ctx contractapi.TransactionContextInterface.GetClientIdentity(), policyContract PolicyContract Ensure: bool or error 1:   policyJSON, err := policyContract.GetPolicy(dataId) 2:   if err != nil 3:   return false, fmt.Errorf(“Failed to get policy: %w”, err) 4:   policy := Policy{} 5:   err = json.Unmarshal([]byte(policyJSON), &policy) 6:   if err != nil 7:       return false, fmt.Errorf(“Parsing policy JSON failed: %w”, err) 8:   attributes, err := ac.getAttributes(ctx) 9:   if err != nil 10:     return false, fmt.Errorf(“Failed to get attributes: %w”, err) 11: if !ac.checkSARule(attributes, policy.SA) 12:   return false, nil 13: if !ac.checkDARule(dataId, policy.DA) 14:     return false, nil 15: if !ac.checkTARule(policy.TA) 16:     return false, nil 17: return true, nil

## 5. Experiment and Analysis

In this section, we rigorously validate the feasibility and effectiveness of our proposed approach through comprehensive experimental evaluation and meticulous result analysis. The validation process comprises four distinct parts: initially, we introduce the experimental environment and describe the parameter settings; next, we present the detailed analysis of the experimental results; subsequently, a thorough security analysis is provided; finally, we engage in a comparative discussion of the functionality in relation to existing approaches.

### 5.1. Experimental Environment

The experimental environment was set up on a computational server and a laptop. The former was used to build a docker version of the HyperLedger Fabric blockchain network simulation environment, while the latter was used to create a hybrid encrypted storage testing environment and simulate client requests. For ease of reference, the main configuration parameters of the two machines and details of the docker nodes are shown in Table 8 and Table 9, respectively. The test data used in this experiment were sourced from real production business data provided by China FAW Group Co., Ltd. in Changchun City.

### 5.2. Experimental Analysis

In this section, we conducted separate performance experiments for the hybrid encrypted storage and the blockchain network. Through using time cost and throughput as two key performance indicators, we comprehensively evaluated the performance of the model and validated the effectiveness of the proposed scheme.

#### 5.2.1. Performance Experiment of Hybrid Encrypted Storage

In order to validate the performance of the hybrid encrypted storage scheme, we employed the JPBC bilinear mapping library for the implementation of the CP-ABE attribute-based encryption algorithm. The AES-128 algorithm was utilized with the CBC mode of operation. To evaluate the time overhead associated with CP-ABE and AES + CP-ABE hybrid encryption across varying data sizes and attribute quantities, we conducted three sets of experiments. Furthermore, we assessed the upload and download performance of the IPFS file system. To mitigate potential experimental errors arising from incidental factors, we averaged the experimental data over five tests for each set.

**Figure 5:**
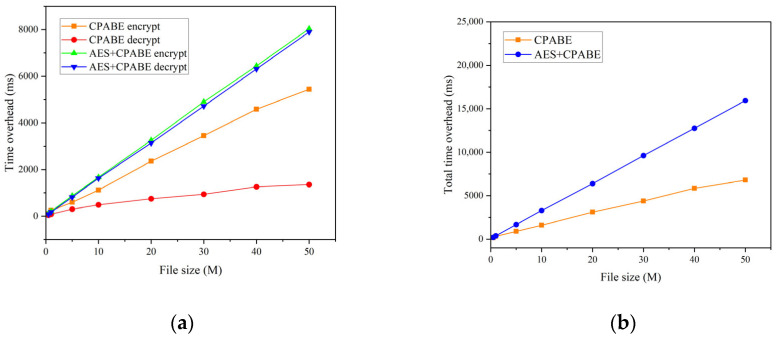
Ten attributes: (**a**) encryption and decryption time overhead; (**b**) overall time overhead.

**Figure 6:**
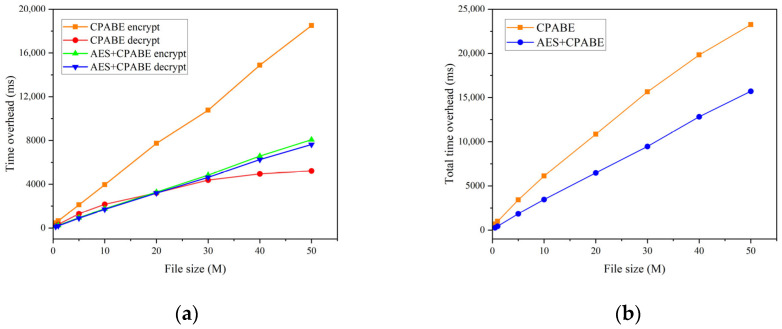
Fifty attributes: (**a**) encryption and decryption time overhead; (**b**) overall time overhead.

**Figure 7:**
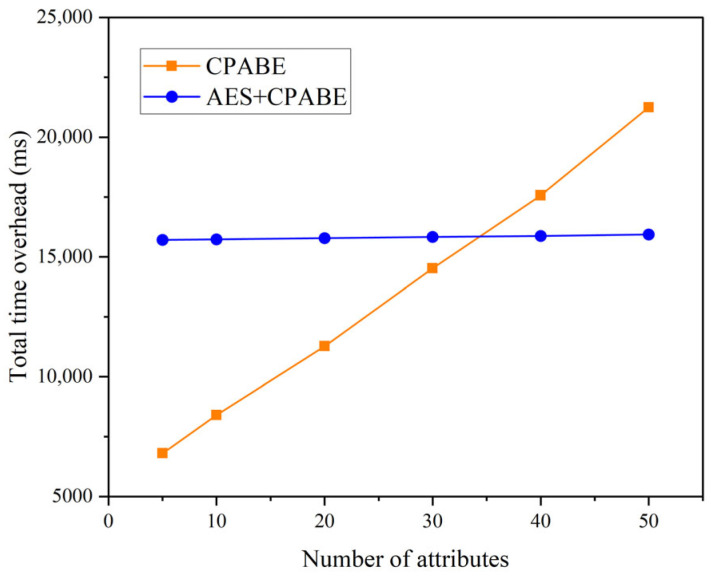
Time overhead for 50 M size.

**Figure 8:**
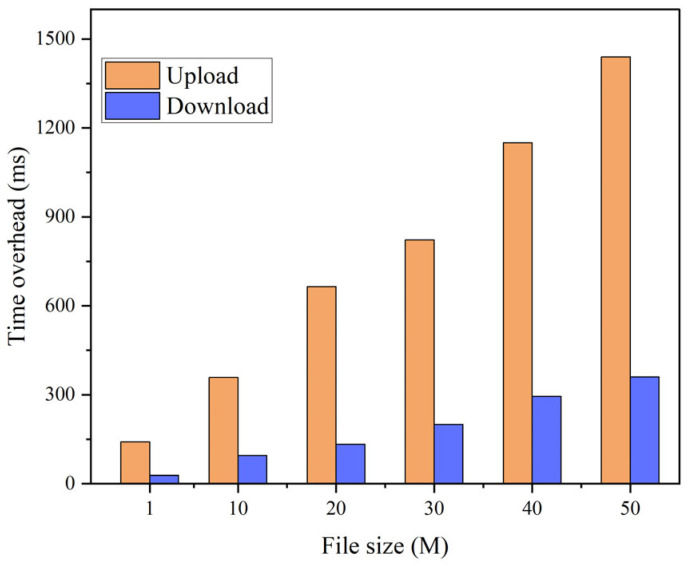
IPFS time overhead.

1. In the initial set of experiments, we maintained two conditions for the total number of attributes: 10 and 50. Correspondingly, the original file sizes were set to 0.5 M, 1 M, 5 M, 10 M, 20 M, 30 M, 40 M, and 50 M, respectively. Our objective was to compare the time overhead incurred during the encryption and decryption processes, as well as the overall time overhead, between CP-ABE and AES + CP-ABE hybrid encryption. The comparative analysis is presented in Figure 5 and Figure 6. Drawing on the experimental findings, it is evident that:
  - In both scenarios, the encryption and decryption times show a consistent linear increase as the file size grows.
  - When utilizing CP-ABE alone, the encryption efficiency surpasses the decryption efficiency significantly. However, in the proposed AES + CP-ABE hybrid encryption scheme, which first employs the AES algorithm to encrypt the original file with equal encryption and decryption efficiency, and subsequently utilizes CP-ABE to encrypt a fixed-length AES key, the time difference in this process becomes negligible due to the shorter key length. Consequently, the overall encryption efficiency becomes nearly equal to the decryption efficiency.
  - Based on the experimental comparisons conducted under the two conditions, it is evident that when the total attribute count is set at 10, employing CP-ABE alone exhibits superior encryption efficiency. However, under the condition of 50 total attributes, the proposed AES + CP-ABE hybrid encryption scheme demonstrates substantial advantages in both encryption and decryption efficiency. Furthermore, as the file size increases, this advantage tends to expand progressively. To further explore the impact of attribute quantity on encryption efficiency, we devised the second set of experiments.
2. In the second set of experiments, we maintained a file size of 50 M and varied the total number of attributes to 5, 10, 20, 30, 40, and 50. Tests were conducted to compare the overall time overhead of CP-ABE and AES + CP-ABE hybrid encryption, as illustrated in Figure 7. Based on the experimental results:
  - When exclusively utilizing the CP-ABE algorithm, the encryption and decryption times exhibit a linear increase as the file size increases. In contrast, the proposed hybrid encryption scheme demonstrates nearly constant time overhead. This is because the encryption and decryption time of the CP-ABE algorithm is combined with the complexity of the access structure. When employing the CP-ABE algorithm for encryption and decryption, calculations and searches are required for each attribute, significantly increasing the workload of bilinear pairing operations and resulting in a linear increase in time with the number of attributes. However, the hybrid encryption scheme combines the advantages of symmetric and asymmetric encryption. It leverages efficient symmetric encryption for data encryption and asymmetric encryption for encrypting the symmetric key. As the number of attributes increases, this hybrid encryption scheme effectively reduces the encryption and decryption time because the key length remains fixed and shorter compared to the original data length. The additional time overhead caused by an increase in attributes can be considered negligible.
  - From the experiments, it can be observed that the encryption time for business data with a size of 50 M can be consistently maintained within 17 s, which fulfills the requirements of practical applications.
3. In the third set of experiments, we varied the sizes of the original files, namely 0.5 M, 1 M, 5 M, 10 M, 20 M, 30 M, 40 M, and 50 M. We conducted tests to assess the upload and download performance of the IPFS file system, with time serving as the evaluation metric. The experimental results are depicted in Figure 8. Based on these findings:
  - The upload and download times in IPFS demonstrate a linear increase as the file size increases, and this trend is significantly influenced by the network speed and bandwidth of the experimental environment.
  - The upload time in IPFS is notably higher than the download time. This disparity can be attributed to the file copying and transmission processes involved in uploading, which consume time and network resources. In cases where the file size is large or the network experiences congestion, the upload time may be prolonged. Conversely, the download process can leverage the distributed nature of the IPFS network to retrieve files from multiple nodes simultaneously, thereby accelerating the download process.

Through a comparative analysis of the time overhead associated with CP-ABE and AES + CP-ABE hybrid encryption across varying data sizes and attribute quantities, our study reveals that our proposed storage scheme offers notable time advantages for handling large datasets and multiple attributes. The computational efficiency of our approach adequately satisfies the demands of practical applications. Regarding space utilization, the adoption of off-chain IPFS distributed storage for storing encrypted files, while storing storage addresses and keys on-chain, enables substantial savings in on-chain storage space. Consequently, this approach indirectly mitigates concerns related to on-chain storage bottlenecks and storage security.

#### 5.2.2. Blockchain Network Performance Experiment

**Figure 9:**
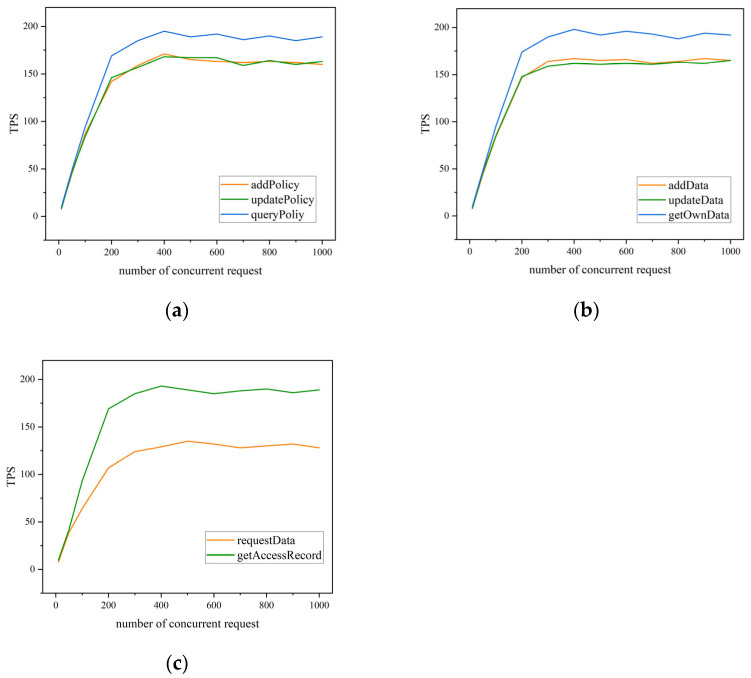
Throughput performance with different concurrent requests: (**a**) StorageContract; (**b**) StorageContract; (**c**) AccessContract and RecordContract.

In order to validate the actual performance of on-chain access control, this experiment leverages Hyperledger Fabric v2.4 as the underlying blockchain development environment. Four organizations, namely Org1 to Org4, are established to simulate different vendors within the supply chain. Each organization consists of two peer nodes and one CA node. The Raft consensus mechanism with Crash Fault Tolerance (CFT) is implemented for transaction ordering. The chaincode is developed using Golang. Following the deployment of the chaincode, HyperLedger Tape is employed to evaluate transaction throughput as a critical performance metric. The test results are shown in Figure 9 for different concurrent request numbers set at 50, 100, 200, 300, 400, 500, 600, 700, 800, 900, and 1000. Based on the experimental results:

- Within the smart contract, query operations generally exhibit higher throughput compared to write and update operations. This can be attributed to the fact that query operations do not necessitate endorsement from nodes, with the computed results directly returned by the nodes. Conversely, write and update operations involve ledger state modification, requiring data replication, consensus confirmation, and consequently consuming more network resources.
- It is evident that system throughput gradually stabilizes after reaching a concurrency of 400. This phenomenon arises due to the hardware resource limitations within the experiment, where the resource utilization of the blockchain network has already reached saturation. Increasing the number of connections no longer enhances concurrent processing capabilities. Nevertheless, each transaction is executed in a queue-like manner without affecting the transactions of individual nodes.
- The requestData() operation exhibits the lowest peak throughput among all operations depicted in Figure 9c. This is attributed to the fact that requesting data necessitates identity and permission verification, entailing greater computational overhead. In comparison to existing research schemes, the attribute information of data requesters is derived from digital certificates instead of being included in the data requests themselves. Although this approach increases the bandwidth requirements for network transmission, as each data request needs to transmit certificates, it may introduce a certain level of network load and transmission latency. However, embedding attribute information into digital certificates enables the separation of attribute details from data requests, thereby reducing the exposure risk of sensitive information during network transmission. Consequently, only authorized entities possess the capability to access shared data, while unauthorized third parties are unable to obtain sensitive information, mitigating the possibilities of attribute data forgery or tampering and providing enhanced security.

### 5.3. Security Analysis

The proposed scheme for supply chain data storage and access control combines secure and efficient encryption algorithms, IPFS, and blockchain technology for multi-layered security mechanisms. Its main goal is to guarantee data confidentiality, auditability, and security while addressing the privacy leakage issues associated with centralized authorization in traditional approaches. This section provides a comprehensive security analysis of our proposed scheme.

**Confidentiality:** In our scheme, data undergoes encryption using the AES symmetric encryption algorithm before being stored in IPFS. AES is widely acknowledged as an efficient and highly secure encryption algorithm. To prevent key leakage during the sharing process, we employ a dual-layer auditable access control model that integrates off-chain attribute encryption and on-chain decentralized contract execution. This approach enables access control and permission management under ciphertext, ensuring that only users who comply with the defined access policies can obtain the necessary keys for data decryption. Consequently, the risk of data leakage during storage and sharing is significantly reduced.

**Integrity and tamper resistance:** Through utilizing IPFS for data storage and combining it with blockchain for sharing, our scheme leverages content addressing and distributed hash tables in IPFS to ensure the integrity of encrypted data. Furthermore, the original data hash values stored on the blockchain guarantee the tamper resistance and integrity of the data throughout the encryption process.

**Eliminating single points of failure:** Our scheme takes advantage of the decentralized nature of IPFS and blockchain through distributing data and keys across multiple nodes. This design significantly enhances the system’s resilience against attacks and mitigates the impact of single points of failure. Even if a node is compromised or experiences a failure, the data remains protected and accessible on other nodes.

**Resilience against attacks:** It can be difficult to prevent collusion attacks altogether, as attackers can use a variety of tactics and strategies to achieve their goals. However, this solution takes the following measures to reduce the risk and impact of collusion attacks: First, it uses decentralized technologies and architectures, thereby reducing trust dependencies between system entities and reducing the likelihood of collusion attacks. Secondly, it uses measures such as encryption technology and authentication using smart contracts, and it reduces the risk of collusion attacks through restricting each entity to access only the data it needs through fine-grained access control policies to reduce the flow of information. Finally, through introducing a data access auditing mechanism, anomalous behavior can be more easily detected, and potential collusion can be stopped in a timely manner. A combination of the above measures will improve the security and trustworthiness of data exchange.

**Auditability:** Smart contracts play a pivotal role in recording data access requests and operation logs, which are subsequently written into the blockchain. This process guarantees the auditability and immutability of the data. In the event of data-related issues, the analysis of the operation records enables the identification of the problem’s source. For malicious users, their data access permissions can be revoked through the use of smart contracts.

**Legal Constraints**: The proposed solution focuses on the relevant legal requirements in the field of data sharing. Considering the conflict between blockchain’s immutability and GDPR, we have adopted a restricted-access consortium blockchain to ensure that only relevant parties can join the network. Additionally, we subject business data to hybrid encryption, which prevents unauthorized users from directly accessing the data, even though it cannot be deleted. Finally, through establishing a flexible and auditable permission management mechanism, we ensure that only authorized users can access specific data. Through auditing capabilities, any unauthorized access attempts can be detected, and access to sensitive data can be restricted, thus complying with GDPR requirements.

### 5.4. Scheme Comparison

To demonstrate the novelty and comprehensiveness of the proposed solution, Table 10 summarizes the functional comparison between our solution and other recent blockchain-based data sharing solutions. Reference [19] adopts smart contracts for simple permission control to ensure system security. However, once data flows change, managing data permissions becomes cumbersome, and storing all data on the blockchain increases storage burden and reduces system efficiency as data volume grows. [27,28] implement flexible access control based on ABAC, but they lack auditable access records, making it difficult to effectively monitor data access and prevent unauthorized actions in a timely manner. The model proposed in [32] is developed based on a public chain, meaning any node can join it, which increases security risks. Ref. [33] adopts an attribute-based access control model for access control, but it does not mention data encryption to enhance data security. Our solution overcomes the above limitations through using IPFS as an off-chain storage system to provide a robust and reliable information storage method. It also leverages the advantages of decentralization, immutability, and traceability provided by the blockchain for data sharing operations. Through the proposed attribute-based dual-layer auditable access control model, it achieves fine-grained and dynamic permission management under ciphertext, effectively reducing the risks associated with unauthorized access and data leakage, thus improving system security and controllability.

## 6. Discussion and Conclusions

In this paper, we propose a blockchain-based secure storage and access control solution for the automotive supply chain ecosystem. Through combining decentralized off-chain IPFS data encrypted storage with on-chain fine-grained controllable data sharing, a secure, efficient, and privacy-protecting decentralized solution for automobile supply chain data storage and sharing has been established. The proposed solution alleviates the storage pressure of blockchain under large-scale data volume and possesses data privacy protection mechanisms. Additionally, we propose, for the first time, an attribute-based dual-layer auditable access control model. This model not only achieves fine-grained and dynamic permission management under ciphertext but also considers privacy and security during the data sharing process. In terms of implementation, we use smart contracts to realize the access control prototype, ensuring that only authorized participants can access business data. We introduce an access log auditing module to ensure the security and legitimacy of data access, helping data owners promptly identify and rectify any anomalous behavior.

Experimental evaluations demonstrate significant time advantages of the proposed hybrid encryption storage scheme under conditions of large-scale data volume and multiple attributes. The access control model maintains high throughput and security in simulated real-world operational scenarios, enhancing the security and privacy of data during the supply chain data flow process. It provides fine-grained access control mechanisms, achieving the intended objectives, and displaying broad prospects.

At the same time, we also recognize the challenges and areas for improvement in this solution. Therefore, we outline future research directions:

1. The proposed solution has been tested with simulated data and analyzed in the laboratory. The next step will involve research and application at China FAW Group Co., Ltd., providing a reference model for the construction of intelligent automotive supply chains in China and other countries.
2. Currently, the attribute authorization authority in the solution adopts a single node. We will further adapt the CP-ABE scheme research to accommodate multiple institutions, dispersing the attribute authorization authorities across different nodes to increase the system’s scalability and fault tolerance, further enhancing its credibility.
3. To further enhance data privacy protection capabilities, the next step will introduce zero-knowledge proof technology in the attribute set verification stage. This technology allows proving that attributes meet the authorization requirements without revealing any information related to the attributes, achieving a higher level of privacy protection.

## Figures and Tables

**Table 1:** Typical business information of each process in the automotive supply chain.

Participant	Process	Link	Shared Business Data	Flow
Component Manufacturer	Processing	Production	Production Batch, Production Date	OEM
		Quality Inspection	Component Name, Batch Number, Inspector, Inspection Date, Inspection Result	OEM
Original Equipment Manufacturer (OEM)	Production	Planning	Production Schedule, Component List	Component Manufacturer
		Production	Batch production, product quantity, production date, inventory data	Logistics Provider, Distributor
		Quality Inspection	Vehicle identification number (VIN), batch number, inspector, inspection date, inspection result	Distributor
Logistics Provider	Transportation	Transportation	Transportation Date, Transportation Route, Quantity of Goods	OEM, Component Manufacturer, Distributor
Distributor	Sales	Sales	Sales Quantity, Sales Time, Sales Location, Sales Price, Customer Information	OEM
		After-sales	After-sales History Records, Maintenance Records, Customer Feedback	OEM, Component Manufacturer

**Table 2:** Main symbols and meanings.

Symbol	The Meaning of Symbol	Symbol	The Meaning of Symbol
DO	Data owner	K	AES key
DR	Data requester	Cid	Unique identifier of the file returned by IPFS
CA	Certificate issuing authority	CK	Ciphertext of AES key after attribute encryption
KMC	Key Management Authority	APK/AMK	System public key/System private key
IPFS	InterPlanetary File System	Att	Attribute
Data	Enterprise’s raw business data	S_Attr_	Set of attributes
Hash_Data_	The hash value of the original data	ASK	User attribute private key
CT	Ciphertext obtained using AES algorithm encryption	EncPolicy	Attribute-based access structure
Cert_attr_	Attribute certificate with identity proof	Policy	Contract-based access control policy

**Table 3:** Attribute definition of SAs.

Name	Definition
factoryName	Factory Name
dept	Department Name
role	Role or Identity Name

**Table 4:** Attribute definition of DAs.

Name	Definition
dataId	Unique Data Identifier
type	Data Type
factoryName	Company Name of the Data Owner
dept	Department Name of the Data Owner
owner	Unique Identifier of the Data Owner’s Identity

**Table 5:** Attribute definition of TAs.

Name	Definition
startTime	Start Time
endTime	End Time

**Table 6:** Examples of policy.

Attributes.json
{“SA”:[{“factoryName”:”LogisticsProvider_B”,”dept”:”InformationDepart”,”role”:”Bob”},{“factoryName”:”Distributor_C”,”dept”:”InformationDepart”,”role”:”Carol”},{“factoryName”:”Distributor_D”,”dept”:”InformationDepart”,”role”:”Dave”}],”DA”:{“dataId”:”PD0001”,”type”:”production_data”,”factoryName”:”VehicleFactory_A”,”dept”:”ProductionDepart”,”owner”:”VehicleFactory_A.ProductionDepart.Alice”},”TA”:{“startTime”:”1679241600”,”endTime”:”1703001600”}}

**Table 7:** Attribute definition of RequestLog.

Name	Definition
Uid	Unique Identifier of the Visitor’s Identity
Result	Access Result
Operation	Access Operation
TimeStamp	Access Timestamp

**Table 8:** Experimental environment.

Device _(1)_	Parameters and Versions	Device _(2)_	Parameters and Versions
CPU	Intel(R) Xeon(R) Gold 5117 CPU @ 2.00GHz	CPU	Apple M1 Pro CPU @ 3.20GHz
Memory	128 GB DDR4	Memory	16 GB DDR5
Hard Disk	2 T	Hard Disk	512 GB
OS	Ubuntu 22.04.2 LTS	OS	macOS Ventura 13.3.1
Docker	v20.10.21	JDK	v11.0.18
Docker-compose	v1.29.2	JPBC	v2.0.0
Golang	v1.18.1	Golang	v1.18.1
Hyperledger Fabric	v2.4.3	IPFS	v0.19.0
Tape	v0.2.5		

In Table 8, _(1)_ represents the configuration of device 1 and _(2)_ represents the configuration of device 2.

**Table 9:** Nodes and number of docker.

Name	Number
Ca	4
Order	3
Peer	8
Fabric-tools	1
Couchdb	8
StorageContract	8
PolicyContract	8
AccessContract	8
RecordContract	8

**Table 10:** Scheme comparison.

Properties	Model					
	[19]	[27]	[28]	[32]	[33]	Our Work
Distributed Storage	√	√	√	√	√	√
ABAC	×	√	√	×	√	√
Encrypted Storage	√	√	×	√	×	√
Auditable	×	×	×	√	√	√
Permission revocable	√	√	√	√	√	√
Blockchain	Fabric	Fabric	Fabric	Ethereum	Fabric	Fabric

## Data Availability

Not applicable.
